# Obstructive Jaundice Due to Primary Choroidal Malignant Melanoma Metastasis: An Unusual Presentation

**DOI:** 10.4103/1319-3767.49011

**Published:** 2009-04

**Authors:** Gaurav Maheshwari, Nairuthya Shivathirthan, Premashish J. Haldar, Dinesh Kamath

**Affiliations:** Department of Surgical Gastroenterology, Jagjivanram Railways Hospital, Maratha Mandir Marg, Mumbai Central, Mumbai - 400 007, India. E-mail: drgauravmaheshwari@gmail.com

**Figure 1:**
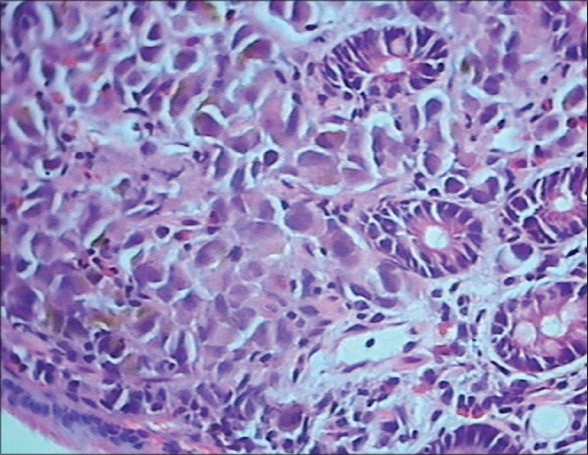
Photomicrograph of malignant melanoma metastasis

Metastatic melanoma of the common bile duct (CBD) is very rare, with only 18 cases reported so far.[1] A 45-year-old male operated 3 years previously for malignant melanoma of the right eye presented to the outpatient department for obstructive jaundice since 25 days with gradual progression. An upper gastrointestinal endoscopy revealed a polypoidal lesion in the second part of the duodenum, which was reported histopathologically as metastasis from the malignant melanoma [Figure 1]. A computed tomography scan of the abdomen showed a complex lesion involving the CBD, gall bladder, and right lobe of the liver, possibly because of metastasis from the primary melanoma of the eye. Poststenting, there was a decrease in his bilirubin levels. The patient was advised immunotherapy but as he was not willing, he was given chemotherapy (Temezolamide).

The first case of a metastatic melanoma to the bile duct was described by Spigelberg in 1895 and the second by Duval in 1908.[2] The melanoma secondaries usually arise from the primary skin lesion but, occasionally, may arise from the primary or metastatic melanoma of the gall bladder. The primary melanoma in patients with gastrointestinal tract metastases is typically located in the extremities (15–57% of the cases) and in the trunk (13–54%) and less frequently in the head and neck (5–33%).[3] There have been no case reports yet of primary choroidal melanoma with metastasis to our knowledge in a review of the literature. Patients with metastatic melanoma to the CBD usually present with progressive painless obstructive jaundice. Obstructive jaundice as the first symptom of the disease due to metastatic melanoma causing ampullary obstruction has been reported only once.[4]

It seems reasonable to perform radical surgical resection in patients with potentially curable disease and isolated deposits in the bile duct. If there is a concurrent metastatic disease elsewhere, it is prudent to adopt a less-aggressive approach in order to relieve obstructive jaundice, such as the bypass procedure or stenting.[5]

Patients with gastrointestinal symptoms and a history of melanoma should be investigated for the presence of gastrointestinal metastases even if the original primary malignancy was diagnosed years before the patient presentation.
